# Composites with Flax and Hemp Fibers Obtained Using Osmotic Degumming, Water-Retting, and Dew-Retting Processes

**DOI:** 10.3390/ma18133200

**Published:** 2025-07-07

**Authors:** Wanda Różańska, Szymon Rojewski

**Affiliations:** Institute of Natural Fibres & Medicinal Plants—National Research Institute, Wojska Polskiego 71B, 60-630 Poznan, Poland; szymon.rojewski@iwnirz.pl

**Keywords:** flax fibers, hemp fibers, osmotic degumming, dew retting, water retting, PE, composite

## Abstract

This study presents the application of flax (*Linum usitatissimum* L.) and hemp (*Cannabis sativa* L.) fibers into composites with polyethylene matrices. The applied fibers were obtained using osmotic, water-retting, and dew-retting processes. The study determined the impact of the fiber extraction method on the properties of the composites obtained from natural filler and polyethylene matrix. These properties included color, tensile strength, thermal stability, adhesion of filler to the polymer, and flammability. It has been shown that the addition of flax and hemp fibers improves the mechanical properties of the composite compared to pure polymer. The tensile strength of the pure polymer samples was 24.64 MPa, while the tensile strength of composites reinforced with flax fibers ranged from 31.26 to 34.45 MPa, and those reinforced with hemp fibers ranged from 31.41 to 33.36 MPa. Studying the composites’ flammability showed that filling them with osmotic degummed hemp fibers reduced the maximum heat release rate by over 34% for hemp compared to pure polymer. This research shows that the composites filled with flax and hemp fibers, regardless of extraction method, are characterized by reduced flammability and improved mechanical properties compared to the pure polyethylene samples.

## 1. Introduction

There is a growing global interest in the use of bast fibers as reinforcement in composites [1,2]. The main advantages of using natural fibers are their low cost, low density, high specific mechanical properties, and biodegradability [3,4]. However, a disadvantage of natural fibers is their low thermal stability and poor water resistance, which weakens the interfacial adhesion between the fibers and polymers [5,6]. Nevertheless, natural fibers as composite reinforcement continue to attract increasing attention.

The literature shows a wide range of methods for obtaining natural fibers for application in composites. For example, studies have used enzymes from the endo-polygalacturonase and pectate lyase groups during the retting process of hemp to improve the mechanical properties of epoxy composites reinforced with hemp fibers [7]. Physical research has included pre-treatment of hemp stems in an autoclave under various steam pressure conditions, as well as a combination of hydrothermal pre-treatment and enzymatic retting. The research discussed in this paper focuses on chemical methods of obtaining fibers and on the use of flax (*Linum usitatissimum* L.) and hemp (*Cannabis sativa* L.) fibers in technical sectors as reinforcement for structural composites [8,9,10,11,12,13]. The effects of different fiber retting treatments on the quality of the resulting composites were also investigated. It was shown that combining physical and biological methods for obtaining fibers resulted in hemp fiber composites with the highest tensile strength and stiffness at 50% fiber volume content—improvements of 3 1% and 41%, respectively, compared to the dew-retted fibers.

Pectate lyase enzymes were also used for the retting of mechanically decorticated flax fibers [14], following the procedure described by Rho [15]. The aim of the study was to compare the properties of dew-retted flax fibers and enzymatically retted fibers in terms of their suitability for composite applications. The authors demonstrated that enzymatically retted fibers showed better shape factor, degree of retting, strength, and thermal stability compared to dew-retted fibers.

Another approach to degumming decorticated flax fibers involved the use of a pectinolytic enzyme (PL-BRI) with lyase activity [16]. The treated fibers were used as reinforcement in composites with vinyl ester resin (Hydropel R037-YDF-40). The study showed that the degree of fiber degumming—that is, the enzyme treatment time—did not affect the quality of the resulting composite. The composite’s strength remained consistent across all samples. However, authors suggested that switching to a more aggressive resin in relation to the fibers might significantly impact composite quality, which warrants further investigation.

In 2018, a study to evaluate flax and hemp fibers obtained through osmotic degumming and traditional dew retting, focusing on their potential for use in composite materials [4], was conducted. This research was part of the international NATURTRUCK project, which aimed to develop composite materials based on biodegradable raw materials such as flax and hemp fibers. The results showed that fibers obtained through osmotic degumming exhibited not only improved adhesion to the polymer matrix but also reduced flammability.

Van de Velde and Kiekens [17] compared the physical, thermal, and mechanical properties of twelve commercially available polymers used as matrices in flax fiber-reinforced composites. The authors concluded that polypropylene (PP) was the best choice, both economically and environmentally. However, they also emphasized the need for the surface modification of flax fibers to improve their adhesion to the polymer matrix. This finding is supported by the results presented in this article, as well as by other researchers [18].

This research focuses on the performance of flax and hemp fibers in composite systems. Fibers were obtained through three different extraction methods, such as osmotic degumming, dew-retting, and warm water-retting. Next, milled fibers were used as fillers in polyethylene composite materials. All fiber-reinforced composites were then evaluated in comparison to polyethylene samples. The choice of polymer matrix was driven by its softening temperature during the composite forming process—below 200 °C—which did not cause fiber degradation. The osmotic, degummed, dew-retted, and warm water-retted fibers were used for the tests. This is significant because the choice of fiber type affects the properties of the obtained composites. The main focus of the work was assessing how the filler influences the microstructure and mechanical properties of composite samples compared to polyethylene samples. The samples were evaluated for their thermal and chemical properties using TGA-FTIR and PCFC analyses, respectively. This research is a continuation of the work previously conducted on assessing the effect of the fiber extraction methods on the quantity and quality of the fiber obtained, as published in the 2023 article entitled ‘The Quantity and Quality of Flax and Hemp Fibres Obtained Using the Osmotic, Water-, and Dew-Retting Processes’ [19]. The study revealed that the properties of the fibers are directly influenced by the extraction method. A comparison of three fiber extraction methods revealed that the osmosis method yields a higher fiber content in the straw than dew-retting or water-retting. Due to the use of these fibers in composite material research, this paper also reports the methodology for obtaining fiber from straw and the methods of evaluating samples.

Konczewicz [20] provided a thorough analysis of the fiber extraction process from straw using the osmotic degumming method, comparing it with dew-retting and water-retting. The paper also examined how the fiber extraction processes influence environmental impact and efficiency, paying particular attention to the new osmotic degumming method.

## 2. Materials and Methods

### 2.1. Materials

Polyethylene HDPE 25055E, DOWTM–polymer matrix (PE), high-density polyethylene.

Flax fibers were obtained from straw by osmotic deglutination, followed by dew-retting and warm water-retting. Flax straw of the Dutch Agatha (NL) variety was collected from the Baird Poland sp. zoo in Bolkow, PL.

Hemp fibers were obtained from straw by osmotic deglutination, followed by dew-retting and warm water-retting. Hemp straw of the Bialobrzeskie (PL) variety was collected from the Lenkon Experimental Station in Steszew, PL.

### 2.2. Degumming Methods of Straw

Three methods of fiber extraction were used to obtain both flax and hemp fibers: osmotic degumming, dew-retting, and warm water-retting.

#### 2.2.1. Osmotic Degumming

Studies on fiber degumming by osmosis were carried out on a large laboratory scale using a device with a periodic raw material loading system, built by the Łukasiewicz Institute for Sustainable Technologies (LIST) in Radom, branch in Lodz, PL.

Both flax and hemp straw were subjected to degumming processes under controlled conditions: a temperature of 30 °C for 72 h for flax and 144 h for hemp, with ultrasound at 20 kHz, a flow speed of water of 30 dm^3^/min, and batch mass 15 kg of flax and 20 kg of hemp. Due to the difference in size of the plants used, whole flax straw was used for testing, while the hemp straw was cut in half. After the degumming process, the straw was subjected to a hydrodynamic rinse using cold water. Then, the excess water was removed using a laboratory wringer. The degummed straw was dried in a laboratory dryer at 60 °C (±5 °C) for 48 h.

#### 2.2.2. Dew-Retting

After harvesting, flax and hemp straw were evenly distributed across the field for dew retting and periodically turned to promote uniform retting. The degree of retting was assessed organoleptically by manually breaking the stems and attempting to remove the woody core (commonly referred to as shives). The retting process was considered complete when the straw could be easily broken and the fibers separated effortlessly from the woody material. Subsequently, the retted straw was gathered from the field and subjected to drying.

#### 2.2.3. Warm Water-Retting

Flax and hemp straw were placed parallel to each other in a specially constructed tank (developed by LIST, PL), secured to prevent displacement, and subsequently submerged in water. The retting process was conducted at a constant water temperature of 30 °C, using a material-to-water ratio of 1:10 (1 kg of straw per 10 dm^3^ of water), with a duration of 72 h for flax and 144 h for hemp. Laboratory experiments were performed using 3 kg of raw material. Following retting, the straw was rinsed with cold water and dried in a laboratory dryer at an air temperature of 60 °C for 48 to 72 h. Mechanical processing of the degummed straw was then carried out using a laboratory-scale scutching unit.

#### 2.2.4. Mechanical Processing of Straw

Following the degumming and retting processes, the flax fibers were extracted mechanically using a laboratory turbine (built by Czech Flax Machinery, CFM, in Merin, CZ). For hemp, a laboratory breaking machine (built by Czech Flax Machinery, CFM, in Merin, CZ) was used first, followed by the laboratory turbine. The breaking machine was used to facilitate processing by the laboratory turbine. 

#### 2.2.5. Milling the Fibers

The obtained long flax and hemp fibers were ground in a RETSCH GmbH (Haan, DE) SM 100 cutting mill using a 1 mm mesh sieve.

### 2.3. Obtaining Composites

For the studies on obtaining composites, a high-density polyethylene polymer was used. Long flax and hemp fibers, obtained from straw by osmotic degumming, dew-retting, and warm water-retting, were subjected to a grinding process using a RETSCH SM 100 cutting mill (built by Retsch GmbH, Haan, DE) with a 1 mm mesh sieve, and were used as the filler for composites.

**Figure 1:**
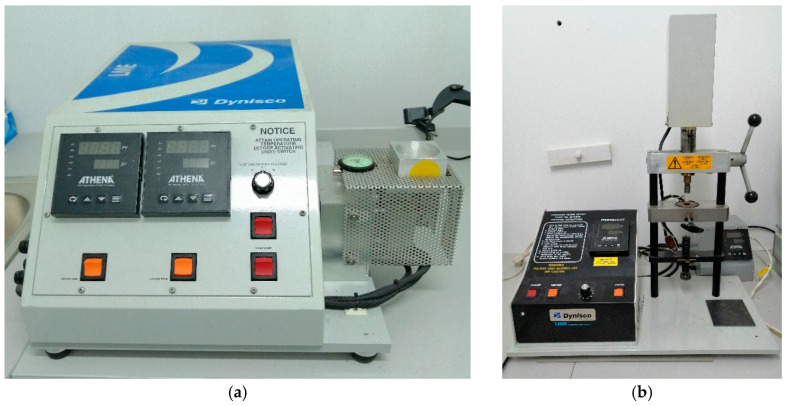
(**a**) Laboratory Mixing Extruder and (**b**) Laboratory Mixing Molder device, built by Dynisco, Franklin, MA, USA.

Before forming the composites, the matrix and filler were dried to a constant mass at 105 °C. The composite filler was mixed at a ratio of 20% flax or hemp fibers and 80% polyethylene using a Laboratory Mixing Extruder (LME) from Dynisco, Franklin, MA, USA, as shown in Figure 1. The extrusion process was carried out under the following conditions: barrel temperature 180 °C and nozzle temperature 165 °C. Next, the samples were formed using a Laboratory Mixing Molder (LMM) from Dynisco, Franklin, MA, USA, as shown in Figure 1. The material was plasticised in the injection moulding machine at a temperature of 190 °C for one minute, while the temperature of the form was 40 °C.

Composite samples were produced in accordance with the ASTM D1708-13 standard [21]. Samples measuring 37 mm in length, 4.7 mm in width, and 3.2 mm in thickness were obtained.

### 2.4. Test Methods

#### 2.4.1. Mechanical Testing

Mechanical tests were carried out on Instron equipment. The UK Model 3119-410 testing machine (built by INSTRON, in Norwood, MA, USA) was used, in accordance with the PN-EN ISO 527-4:2000 standard (Plastics): Determination of mechanical properties in static tension—Test conditions for isotropic and orthotropic fiber-reinforced plastic composites [22].

The tests were performed in controlled climatic conditions, with a temperature of 20 °C and an air humidity level of 65%. The length between the clamps was set to 20 mm, and a constant elongation increase of 10 mm/min was applied until the sample broke.

#### 2.4.2. Microscopic Analyses

Microstructural observations of longitudinal and cross-sectional views of flax and hemp fibers were conducted using a scanning electron microscope (SEM), model Hitachi S-3400N (Hitachi, Ltd., Tokyo, Japan), operated in high vacuum mode with a secondary electron (SE) detector. Prior to imaging, the fiber surfaces were coated with a thin layer of gold. Observations were performed at a magnification of ×250, with a table height set at 20 mm and an accelerating voltage of 20 kV, enabling the visualization of characteristic surface features of the fibers.

#### 2.4.3. Combined Analysis TGA-FTIR

Thermogravimetric analysis (TGA) was carried out using a Q50 analyzer (TA Instruments, New Castle, DE, USA). Approximately 15 mg of the test sample was subjected to heating from 30 °C to 700 °C at a constant rate of 10 °C/min under a nitrogen atmosphere, with a continuous gas flow rate of 90 mL/min.

Simultaneously, the gaseous decomposition products released during TGA were analyzed by Fourier Transform Infrared Spectroscopy (FTIR). This analysis was performed using a Thermo Fisher Scientific iZ10 system (Madison, WI, USA). FTIR spectra were recorded at a resolution of 4 cm^−1^ across a spectral range of 600 to 4000 cm^−1^, with a scan rate of 8 scans per second.

#### 2.4.4. Flammability Tests

Flammability characteristics were examined using a Pyrolysis Combustion Flow Calorimeter (PCFC) manufactured by FTT, UK. The procedure followed the ASTM D7309-2007 standard [23]. The pyrolysis process was conducted over a temperature range of 75–750 °C at a heating rate of 1 °C/s, followed by combustion at 900 °C. The gas flow consisted of an O_2_/N_2_ mixture at a ratio of 20/80 cm^3^/min. Test samples weighed between 3 and 4 mg. Key parameters measured included the maximum heat release temperature (Tmax) and the maximum heat release rate (HRRmax).

#### 2.4.5. Statistical Analysis

Statistical evaluation was performed to identify significant differences between fiber samples. One-way analysis of variance (ANOVA) was applied, followed by Tukey’s Honest Significant Difference (HSD) post hoc test. A significance threshold of *p* < 0.05 was used to determine statistically meaningful differences.

## 3. Results and Discussion

**Figure 2:**
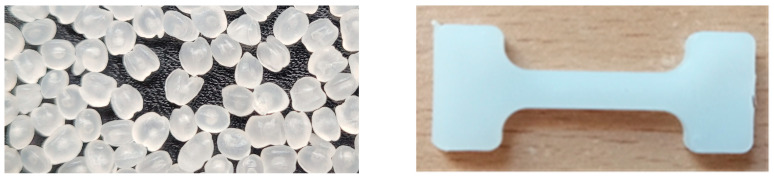
View of raw materials (PE) and polyethylene sample (PE_C).

For the studies on obtaining composites, a polyethylene matrix was used in combination with flax or hemp fibers to plasticize the materials below the degradation temperature of fibers of 200 °C, as shown in Figure 2. According to the authors of [6,17], the decomposition temperature of bast fibers determines whether the matrix can be used.

**Figure 3:**
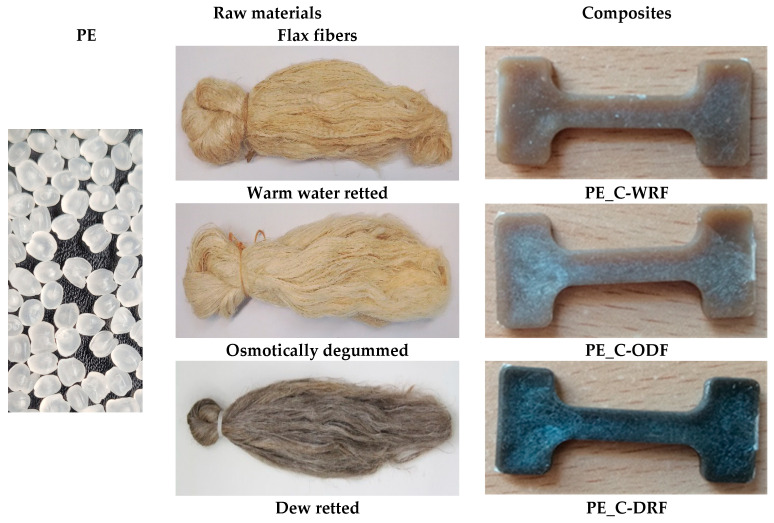
View of raw materials and flax fiber-reinforced composites.

**Figure 4:**
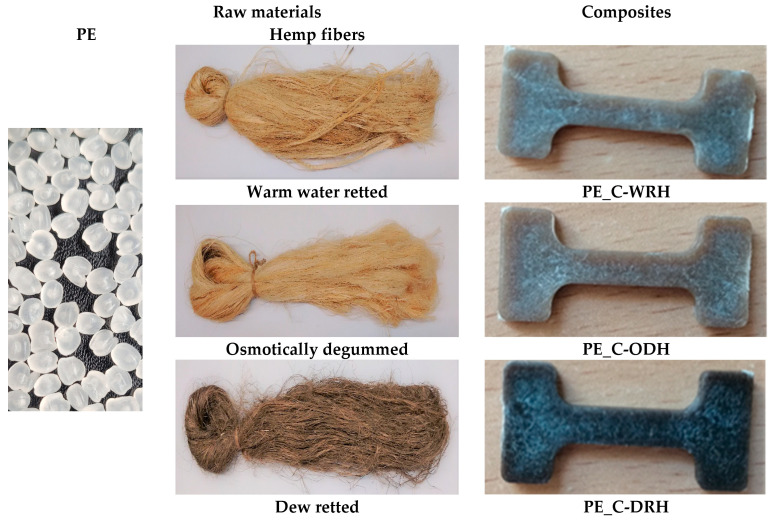
View of raw materials and hemp fiber-reinforced composites.

Composites were formed using an extruder with a polyethylene matrix and a filling of flax or hemp fibers, which were obtained after three extraction processes: dew-retting, warm water-retting, and osmotic degumming, as shown in Figure 3 and Figure 4. The fiber distribution ranged from 0.7 to 1.7 mm for flax fibers, with an average value of 1.2 mm. For hemp fibers, the range was from 0.8 to 1.5 mm, with an average value of 1.1 mm.

The fiber-reinforced composites were compared with the samples obtained with a 100% polyethylene matrix, as shown in Figure 2, Figure 3 and Figure 4.

To improve fiber adhesion to the polymer matrix, the obtained fibers were subjected to a drying process to eliminate humidity. As reported by Abdul Moudood et al. [24], flax fibers have the ability to absorb water and moisture, thereby affecting the mechanical properties of the resulting composites.

Previous studies have shown that the hygroscopicity of flax fibers was 8.80–9.44% at 65% humidity, 9.81–10.85% at 100% humidity, while for hemp fibers it was 11.48–11.94% and 13.14–13.96%, respectively [19].

It was demonstrated that composites containing flax or hemp fibers are brown, whereas pure polymer is white. It was also shown that the method used to obtain the fibers affects the color of the composite. Composites reinforced with osmotic degummed and warm water-retted fibers were characterized with a light brown color, while those with dew-retted fibers were darker.

As demonstrated in a previous publication, the method used to extract the fiber is the key factor determining its color [19].

In the water-retting process, bacteria such as *Bacillus amylobacter*, *Bacillus felsineus*, *Granulobacter pectinovorum*, *Clostridium felsineum,* and *Bacillus comesii rossi* cause the fermentation of pectin substances and the separation of the woody stem parts from the fibers [25,26,27,28,29,30]. However, in the osmosis process, fiber extraction is based on the use of physical phenomena, diffusion and osmosis, occurring in contact with water inside fiber plants, which makes it possible to extract fibers from plants without affecting the natural characteristics of the separated fiber. Both in the water-retting and osmotic degumming methods, the obtained fibers are characterized by a light color, consistent with the raw material used [20].

In the dew-retting method, fungi such as *Cladosporium herbarum*, *Mucor* sp., *Rhizopus* sp., and *Epicoccum nigrum* decompose pectins, which are commonly known as plant glues. This causes the color of the flax or hemp straw to change from yellow-green to steel grey [28,29].

### 3.1. Mechanical Tests

Mechanical testing of composites has shown that those reinforced with flax or hemp fibers, regardless of the production method, have a higher tensile strength: 19–28% for flax and 21–25% for hemp compared to the polyethylene sample. This has been statistically confirmed.

The composite reinforced with flax fiber obtained in the osmotic degumming process had the highest tensile strength value (34.45 MPa). In contrast, the composite reinforced with hemp fibers obtained in the field-retting process had a tensile strength value of 33.36 MPa, as shown in Table 1.

The greatest difference in the study’s results was observed for tensile stress. For flax, the difference between the samples reinforced with osmotically degummed and the dew-retted fibers was 3.19 MPa, which was statistically confirmed. The differences were not statistically confirmed for composites reinforced with osmotically degummed flax fibers and warm water-retted flax fibers (tensile stress difference of 1.71 MPa).

For all fiber-reinforced hemp samples, the differences in tensile stress were less than 2 MPa and were therefore not considered statistically significant.

This research has shown that flax and hemp fibers can be used as composite reinforcement, regardless of the extraction method.

In their studies, Li et al. [31] showed that using flax fibers containing 58% shives in LDPE polyethylene composites allows a tensile strength of about 13.4 MPa to be obtained without surface modification, and a strength of 13.7 to 14.3 MPa for modified fibers. For biocomposites with HDPE, the tensile strength is 19.5 MPa without fiber modification, and between 20.1 and 20.5 MPa for modified fibers.

Velde and Kiekens [17] compared the physical, thermal, and mechanical properties of low-density polyethylene (LDPE) and high-density polyethylene (HDPE). They found that both polymers are suitable for use as a matrix material in flax fiber-reinforced composites. LDPE has a lower density of 0.910–0.925 g/cm^3^, which makes the composite lighter.

In addition, studies have shown that polyethylene samples have a significantly higher elongation at break parameter of around 94–96%, compared to composites reinforced with flax and hemp fibers. This has been statistically confirmed. However, no significant differences were observed in composites filled with fibers, regardless of the extraction method. This parameter is important in assessing performance as it reflects the material’s ability to deform and its strength during stretching. Therefore, composites reinforced with fibers are characterized by lower elasticity and ductility, and thus show lower impact resistance compared to polyethylene samples.

Furthermore, a higher elastic modulus was observed for fiber-reinforced composites, about 29–38% for flax and 41–46% for hemp compared to polyethylene samples. This was statistically confirmed. Significant differences were observed in fiber-reinforced composites filled with osmotic degummed and dew-retted flax fibers compared to water-retted flax fibers. Similarly, significant differences were observed between osmotic degummed and water-retted fibers compared to dew-retted fibers. This parameter indicates the material’s stiffness and resistance to elastic (i.e., nonpermanent) deformation. Studies have shown that fiber-reinforced composites are stiffer and more difficult to deform under force. This demonstrates the material’s greater ability to deform under an applied force and then return to its original shape once the force is removed.

### 3.2. Microscopic Tests

**Figure 5:**
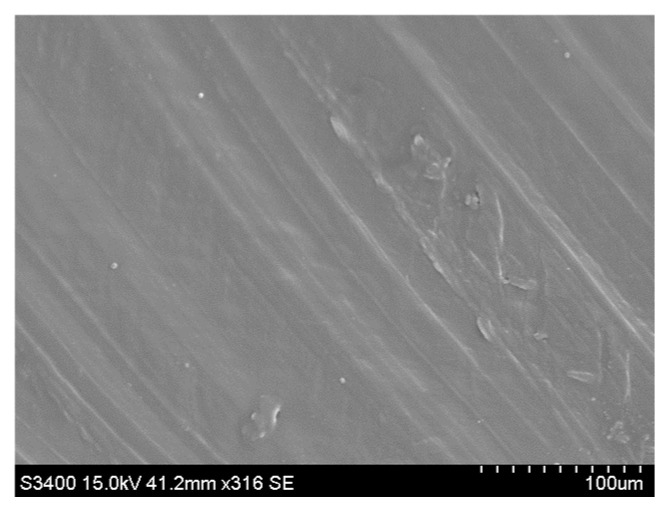
View of composite (PE_C) breakthrough. Scanning electron microscope (SEM), 250 magnification.

**Figure 6:**
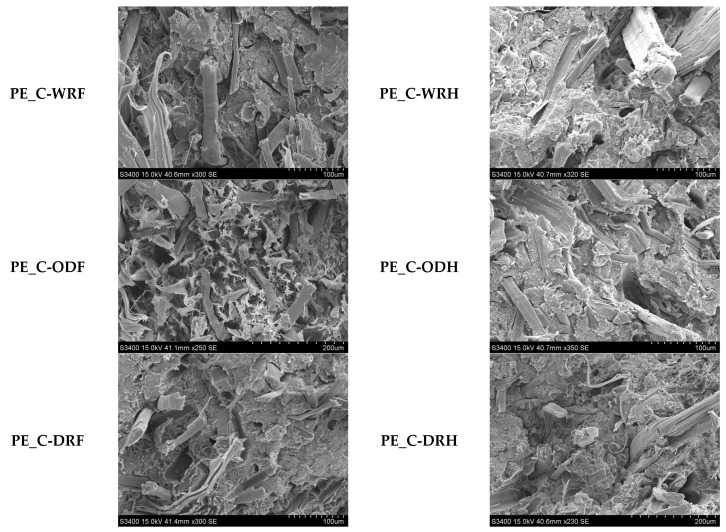
View of the fiber-reinforced composites breakthrough. Scanning electron microscope (SEM), 250 magnification.

Microscopic tests have shown that empty spaces and fiber holes can be observed between the flax or hemp fibers and the polymer, regardless of the fiber extraction method, as shown in Figure 5 and Figure 6.

Microscopic examination of tested samples revealed that the fracture surface of all composites was rough and inhomogeneous, with numerous elongated, cylindrical fibers visible, as shown in Figure 6. SEM images showed that flax fiber-reinforced composites have a more inhomogeneous surface than hemp fiber-reinforced composites. This is reflected in the results of the mechanical tests, which showed that composites reinforced with flax or hemp fibers have improved mechanical properties compared to polyethylene samples.

Moreover, when comparing the samples based on the type of fiber treatment, the composite reinforced with flax fibers extracted via osmotic degumming exhibited the highest surface inhomogeneity. In contrast, the composites reinforced with water-retted flax fibers demonstrated the most uniform surface morphology. In the case of hemp fiber-reinforced composites, the influence of the extraction method on surface uniformity was less significant.

This effect can be explained by the extraction of fibers from the matrix when samples were prepared for testing, which was related to the poor adhesion of the fibers to the polymer matrix [19]. Only the further modification of the fibers with silanes, for example, will effectively increase the interfacial adhesion between the filler and the polymer [32,33,34,35]. This results in the formation of a hydrophobic thin coating on the fiber surface and the creation of a chemical bond between the fibers and the matrix [36,37,38].

### 3.3. Combined Analysis TGA-FTIR

**Figure 7:**
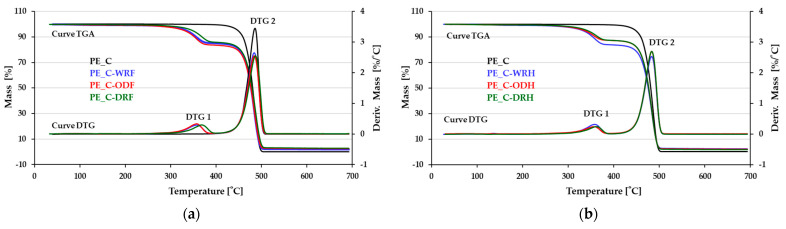
Thermogram of composites. Fiber-reinforced composite samples. (**a**) Flax and (**b**) hemp.

During the thermal decomposition of polyethylene (PE), a single-stage decomposition of the sample was observed within the temperature range of 400–508 °C. By contrast, a two-stage thermal decomposition was observed for fiber-reinforced composite samples, starting at around 240 °C, as shown in Figure 7.

For all tested fiber-reinforced composite samples, the first stage of decomposition occurs at temperatures between 236 and 390 °C, which is related to the degradation of the filler (i.e., the fibers themselves, as shown in Table 2). The second stage of decomposition for composites filled with all three types of tested fibers occurs at temperatures between 404 and 512 °C, which is related to the degradation of the matrix (i.e., polyethylene). Decomposition of the fibrous material in the composite occurs at a higher temperature than that of the fiber itself, as detailed in the authors’ previous publication, ‘The Quantity and Quality of Flax and Hemp Fibers Obtained Using the Osmotic, Water-, and Dew-Retting Processes’ [19]. Based on this, it can be concluded that the fiber–matrix interaction is strong enough to alter the thermal properties of the composite’s primary component by 60–70 °C.

**Figure 8:**
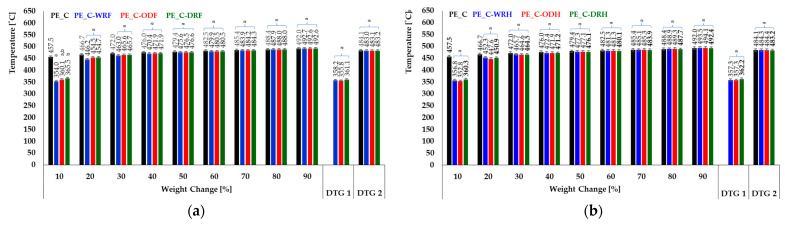
Mass change of composites (pure and fiber-reinforced samples) during pyrolysis. (**a**) Flax and (**b**) hemp. Results are presented as mean ± standard deviation (SD), with a sample size of n = 3. No assigned letters indicate statistically significant differences between group means. The designation ‘a,b’ refers to the comparison of statistics for flax, and ‘a’ hemp.

Based on the data from the above thermograms, it can be concluded that, regardless of how the fibers are obtained as a composite filler, they undergo thermal degradation within a similar temperature range, as shown in Figure 8. However, this has not been statistically confirmed. Therefore, the method used to separate the fibrous material from the rest of the straw appeared not to affect the thermal resistance of the fibrous composites. The differences in plateau level visible between stages I and II can be related to small differences in fiber content in the composite. As thermal decomposition of natural fibers occurs at lower temperatures than synthetic polymers, as can be seen in the composite thermograms, this indicates that composite degradation begins at a lower temperature than polyethylene samples. This information is confirmed by the DTG curves, which describe the rate of mass loss on the TGA thermograms. The derivatives’ maxima are located at the same temperatures, showing no differences for fibers obtained by different methods. Thermal curves with single-stage decomposition derived from polyethylene have one DTG maximum. As expected, two maxima can be observed in the DTG curves for two-stage decomposition. In the event of differences in the amount of solid residue remaining after pyrolysis, all fiber-reinforced samples are characterized by a similar level of residue, i.e., 2.29–2.79% for flax and 2.26–2.93% for hemp. Therefore, the amount of gases released is also similar and not statistically significant, as shown in Table 2.

Infrared studies of the gases released during the thermal decomposition of flax- or hemp fiber-reinforced composites have shown that, in the first stage of decomposition (the DTG 1 peak), which is associated with fiber degradation, bands appear corresponding to the vibrations of functional groups such as OH, CO, C=O, C=C, COO, CH, and CH_3_.

The detailed analysis of the spectra for flax and hemp fiber-reinforced composites, as shown in Figure 6 for the DTG1 peak, revealed the release of gases, as shown in Table 3.

All of the gases, as shown in Table 3, originate from the thermal decomposition of the fiber in fiber-reinforced composites. This is confirmed by fiber pyrolysis studies published in 2023, regardless of the fiber acquisition method [19]. Only methane could not be confirmed in the tests, which can be explained by its low concentration in the composite samples. As reported by other authors, the pyrolysis of the fibers involves the decomposition of the fibers with the release of water vapor, carbon dioxide, carbon monoxide, formic acid, acetic acid, methanol, formaldehyde, and phenol [39,40,41,42,43,44].

In the second stage of decomposition (DTG peak 2) of composites filled with flax or hemp fibers, as well as in the first stage of the degradation of pure polymer, some bands mainly correspond to the vibrations of functional groups: CH_3_, CH_2_, =CH, and a smaller number of bands corresponding to vibrations of functional groups: OH, C=O, C-O, and CO_2_.

**Figure 9:**
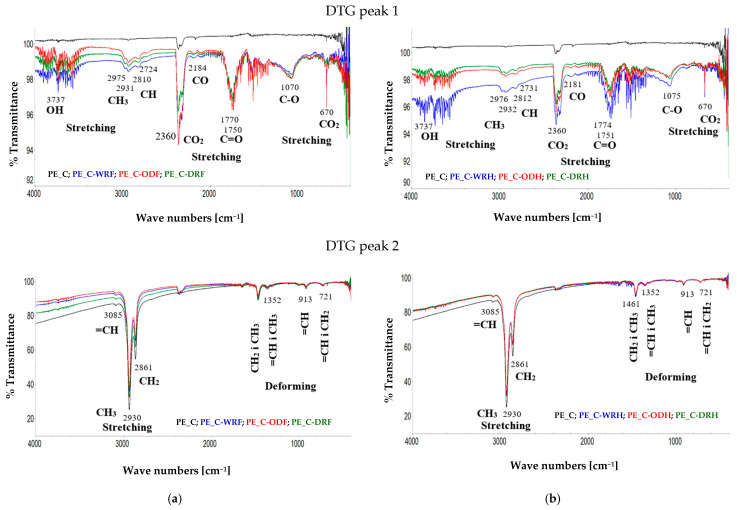
FTIR spectra of released gases during the pyrolysis of composites: (**a**) flax, (**b**) hemp.

The detailed analysis of the spectra revealed the release of mainly aliphatic hydrocarbons (C1-C5), both saturated and unsaturated, as shown in Figure 9 for DTG peak 2. For saturated hydrocarbons, the stretching vibrations of the CH_3_ bond are visible at a wavelength of 2930 cm^−1^, and the stretching vibrations of the CH_2_ bond are visible at a wavelength of 2861 cm^−1^. The deformation vibrations of the CH_3_ and CH_2_ bonds are visible at a wavelength of 1461 cm^−1^. The gases mentioned were also identified in the fibers, regardless of the method used to obtain them [19]. Methane was the only gas not confirmed in the tests, which can be explained by its low concentration in the composite sample.

In the case of unsaturated hydrocarbons, stretching bands appear at a wavelength of 3085 cm^−1^ for the =CH bond, as well as out-of-plane deformation bands at a wavelength of 913 cm^−1^. By contrast, visible in-plane deformation bands originating from the =CH and CH_3_ bonds at 1352 cm^−1^, as well as deformation vibrations originating from the =CH and CH_2_ bonds at 721 cm^−1^, are common to both saturated and unsaturated hydrocarbons.

In addition to hydrocarbons, analysis of the infrared spectrum revealed weak signals originating from water (OH bond stretching vibrations at a wavelength of 3737 cm^−1^), carbon dioxide (CO bond stretching vibrations at wavelengths of 2360 cm^−1^ and 671 cm^−1^), and bands characteristic of the carbonyl group (C=O) at a wavelength of 1642 cm^−1^. The presence of these gases during the pyrolysis of the fiber is understandable and related to its decomposition. However, in the case of the pure polymer, the formed gases may originate from resin impurities and unidentified gases released during the pyrolysis of PE. According to Klein [45], pyrolysis gases containing mainly aliphatic hydrocarbons (C_1_-C_5_) in amounts ranging from 98.7% to 99.6% (both saturated and unsaturated) and unidentified gases in amounts ranging from 0.4% to 1.3% are formed during the pyrolysis of polyethylene. 

### 3.4. Flammability Tests

**Figure 10:**
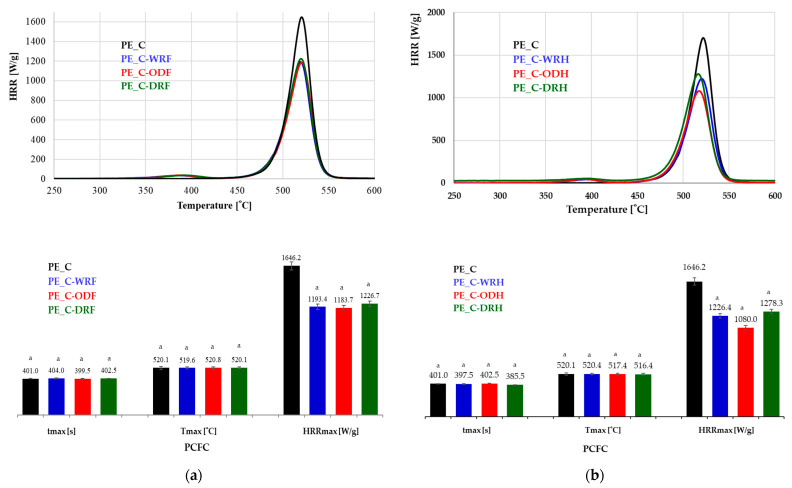
Flammability results of composites: (**a**) flax, (**b**) hemp. Results are presented as mean ± standard deviation (SD), with a sample size of n = 3. No assigned letters indicate statistically significant differences between group means. The designation ‘a’ refers to the compari-son of statistics for flax and hemp.

Flammability tests of fiber-reinforced composites have shown that adding flax and hemp fibers reduces the combustion intensity of the resulting composite compared to the pure polymer, as shown in Figure 10, a finding that has been statistically confirmed.

Analysis of the results for flax showed that the maximum heat release rate (HRR_max_) was achieved for the pure polymer at 1646.181 W/g, which was over 28% higher than for the composite reinforced with osmotically degummed fibers. The value for the latter was the lowest at 1183.673 W/g, a statistically significant difference. The heat release temperature of the polymer was similar to that of the composite reinforced with flax fibers, i.e., ±520 °C. The difference in time was 1.5 s longer compared to the osmotically degummed fiber-reinforced composite, and 3 s shorter compared to the water-retted fiber-reinforced composite. However, these data have not been statistically confirmed.

In the case of composites filled with hemp fibers, the maximum heat release rate (HRR_max_) for the pure polymer was over 34% higher than for the composite reinforced with osmotically degummed fibers, for which the HRR_max_ value was the lowest at 1080.2 W/g. This difference was statistically significant. The polymer’s heat release rate temperature was 2.7 °C lower than that of the hemp fiber-reinforced composite, 1.5 s longer than that of the osmotically degummed fiber-reinforced composite, and 17 s longer than that of the dew-retted fiber-reinforced composite. The differences in the results for heat release time and maximum combustion temperature obtained for all the tested samples were not statistically significant.

In both cases, the effect of the fiber extraction method on the maximum heat release rate of both flax and hemp fibers as a filler, any significant differences were found. Referring to previous studies on the fibers themselves [19], the osmotic degumming method was found to significantly reduce the flammability of hemp fibers only. In other cases, no substantial differences were observed.

The results indicate that adding flax and hemp fibers reduces the flammability of the composite, regardless of the extraction method applied. This increases the composite’s fire resistance compared to pure polymer, a fact that was statistically confirmed. Conversely, the differences in the burning rate of the composites depending on the method used to obtain the fibers showed that flax and hemp fibers obtained using osmotic degumming had the lowest value compared to those obtained using dew- or warm water-retting. However, these differences were not statistically significant. Furthermore, Kandola and Horrocks [46] reported that the increased burning rate of composites is influenced by poorer adhesion between the fibers and the polymer matrix. Therefore, it can be concluded that fibers obtained using osmotic degumming exhibit greater adhesion to the polymer matrix than fibers obtained using other methods.

## 4. Conclusions

The results of this study proved that the application of flax and hemp fiber fillings obtained using osmotic degumming, dew-, or water-retting into a polyethylene matrix improves the mechanical properties and reduces the flammability of the composites compared to the pure polymer.

The tests carried out for all tested fibers showed that:

1. The addition of flax and hemp fibers improves the mechanical properties of the composite compared to pure polymer. The tensile stress ranged from 31.26 to 34.45 MPa for flax fiber-reinforced composites, ranged from 31.41 to 33.36 MPa for hemp fiber-reinforced composites, and 24.64 MPa for pure polyethylene samples. Therefore, flax and hemp fibers can be used for reinforcement in composites.
2. Regardless of the method used to obtain them, the adhesion of flax and hemp fibers to the polymer matrix is poor when they are used as fillers, as can be seen in SEM photos.
3. The thermal stability of composites with polyethylene/flax or hemp fibers does not depend on the method used to obtain the fibers. However, the addition of flax and hemp fibers themselves causes a deterioration in the thermal stability of the composite compared to pure polymer. Polyethylene undergoes thermal decomposition at temperatures between 400 and 520 °C, whereas the composite with flax or hemp fiber decomposes at temperatures between 230 and 520 °C.
4. In the thermal decomposition process of the composite with the addition of flax and hemp fibers, a two-stage decomposition is observed. In the first stage, the fibers decompose, while in the second stage, the polymer decomposes. In the first stage, gases such as carbon dioxide, carbon monoxide, water vapor, acetic acid, formic acid, and formaldehyde were identified, regardless of the method of obtaining fibers. In the second stage, regardless of the method of obtaining the fibers, gases were identified in the form of aliphatic hydrocarbons (C_1_-C_5_), both saturated and unsaturated, carbon dioxide, water vapor, and gases containing a carbonyl group.
5. Flax and hemp fibers used as fillers to reinforce pure polyethylene composites reduce flammability and thus increase their fire resistance compared to pure polymers. The same effect can be observed for the fibrous material in the composite, which decomposes at a higher temperature than the fiber itself. For all the tested composites, the sample filled with osmotic degummed hemp fibers exhibited the lowest flammability.
6. The flax and hemp fibers—whether osmotically degummed, water-retted, or dew-retted fibers—can be successfully used as biodegradable fillers in composite materials based on polymer matrices. Moreover, implementing various modifications may improve the compatibility of the fibers with diverse matrices. The resulting modified fibers could find applications in the production of packaging materials, filaments for 3D printing, and in the automotive sector, among others.

## Figures and Tables

**Table 1:** Results of mechanical tests on composite samples containing natural fibers.

Composite Type	Tensile Stress		Elongation at Break		Elastic Modulus	
	[MPa]	SD	[%]	SD	[MPa]	SD
PE_C	24.64	1.10	218.61	33.95	652.58	19.72
FLAX						
PE_C-WRF	32.74 ^a,b^	1.80	12.43 ^a^	1.57	918.34	21.17
PE_C-ODF	34.45 ^b^	1.24	10.54 ^a^	0.87	1060.09 ^a^	30.64
PE_C-DRF	31.26 ^a^	1.52	10.11 ^a^	3.13	1021.68 ^a^	74.10
HEMP						
PE_C-WRH	31.41 ^(a)^	1.01	7.01 ^(a)^	0.33	1217.77 ^(a)^	20.68
PE_C-ODH	32.90 ^(a)^	1.49	7.32 ^(a)^	0.62	1213.31 ^(a)^	44.69
PE_C-DRH	33.36 ^(a)^	1.14	8.97 ^(a)^	0.80	1113.13	45.26

Results are presented as mean ± standard deviation (SD), with a sample size of n = 4. No assigned letters indicate statistically significant differences between group means. The designation ‘a,b’ refers to the comparison of statistics for flax/PE_C, and ‘(a) hemp/PE_C’.

**Table 2:** The thermal properties of composites.

Composite Type	Decomposition of Fibers Stage			Decomposition of PE Stage			Residual Mass at ±510 °C
	T_Int._	T_Fin._	Mass Loss	T_Int._	T_Fin._	Mass Loss	
	[°C]	[°C]	[%]	[°C]	[°C]	[%]	[%]
PE	-	-	-	400 ^a (a)^	508 ^a (a)^	99.26	0.06
FLAX							
PE_C-WRF	245 ^a^	387 ^a^	13.82 ^a^	409 ^a^	512 ^a^	82.71 ^a^	2.30 ^a^
PE_C-ODF	240 ^a^	387 ^a^	15.28	405 ^a^	512 ^a^	80.76 ^a^	2.79 ^a^
PE_C-DRF	238 ^a^	389 ^a^	13.73 ^a^	404 ^a^	510 ^a^	83.05 ^a^	2.29 ^a^
HEMP							
PE_C-WRH	236 ^(a)^	389 ^(a)^	14.99	406 ^(a)^	511 ^(a)^	80.75 ^(a)^	2.93 ^(a)^
PE_C-ODH	236 ^(a)^	385 ^(a)^	11.87 ^(a)^	406 ^(a)^	510 ^(a)^	84.59 ^(a)^	2.53 ^(a)^
PE_C-DRH	243 ^(a)^	390 ^(a)^	11.83 ^(a)^	407 ^(a)^	510 ^(a)^	84.90 ^(a)^	2.26 ^(a)^

Results are presented as mean ± standard deviation (SD), with a sample size of n = 3. No assigned letters indicate statistically significant differences between group means. The designation ‘a’ refers to the comparison of statistics for flax/PE_C, and ‘(a) hemp/PE_C’.

**Table 3:** The list of identified gases during thermal decomposition of fiber-reinforced composites.

Compound Identified	Molecular Formula	Functional Group	Wave Number cm^−1^
Water vapor	H_2_O	OH	3737
Carbon dioxide	CO_2_	CO	2360 and 671
Carbon monoxide	CO	CO	2184
Acetic and Formic acids	CH_3_COOH and CHOOH	OH C=O C-O -CH_3_ -CH	3570 1751–1750 1075–1070 2979–2976 2932-2931
Formaldehyde	CHOH	C-H C=O	2812–2810 and 2731–2724 1774–1770

## Data Availability

The original contributions presented in this study are included in the article. Further inquiries can be directed to the corresponding author.

## Abbreviations

The following abbreviations are used in this manuscript:

DTG	Derivative Thermogravimetry
FTIR	Fourier Transform Infrared Spectroscopy
HRRmax	Maximum heat release rate
INFMP-NRI	Institite of Natural Fibres & Medicinal Plants—National Research Institute
LIST	Łukasiewicz, Institute for Sustainable Technologies
LME	Laboratory Mixing Extruder
LMM	Laboratory Mixing Molder
PCFC	Pyrolysis and Combustion Flow Calorimeter
PE	Polymer, High-Density Polyethylene (HDPE)
PE_C	Polyethylene sample
PE_C-WRF	Water-Retted Flax Fiber-Reinforced Composite Sample
PE_C-WRH	Water-Retted Hemp Fiber-Reinforced Composite Sample
PE_C-ODF	Osmotically Degummed Flax Fiber-Reinforced Composite Sample
PE_C-ODH	Osmotically Degummed Hemp Fiber-Reinforced Composite Sample
PE_C-DRF	Dew-Retted Flax Fiber-Reinforced Composite Sample
PE_C-DRH	Dew-Retted Hemp Fiber-Reinforced Composite Sample
SD	Standard deviation
TGA	Thermogravimetric analysis
